# Correction: Surface hardness and flammability of Na_2_SiO_3_ and nano-TiO_2_ reinforced wood composites

**DOI:** 10.1039/c9ra90075f

**Published:** 2019-10-30

**Authors:** Edita Garskaite, Olov Karlsson, Zivile Stankeviciute, Aivaras Kareiva, Dennis Jones, Dick Sandberg

**Affiliations:** Wood Science and Engineering, Department of Engineering Sciences and Mathematics, Luleå University of Technology Forskargatan 1 SE-931 87 Skellefteå Sweden edita.garskaite@ltu.se +46-72-2332094; Institute of Chemistry, Faculty of Chemistry and Geosciences, Vilnius University Naugarduko 24 Vilnius LT-03225 Lithuania

## Abstract

Correction for ‘Surface hardness and flammability of Na_2_SiO_3_ and nano-TiO_2_ reinforced wood composites’ by Edita Garskaite *et al.*, *RSC Adv.*, 2019, **9**, 27973–27986.

The authors regret that Fig. 1–4 were shown in an incorrect order in the original manuscript. Fig. 1 in the original manuscript should be Fig. 3, Fig. 2 should be Fig. 4, Fig. 3 should be Fig. 1, and Fig. 4 should be Fig. 2. The correct order of the figures is shown below.

**Fig. 1:**
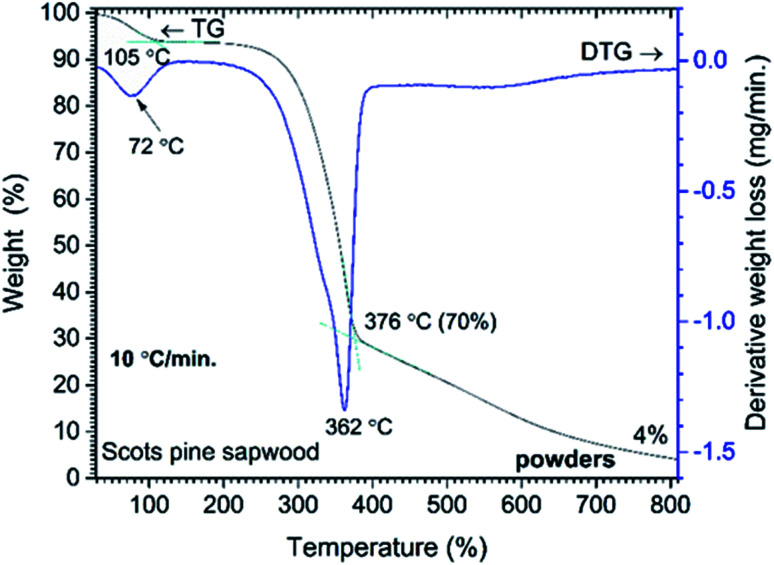
Percent weight-loss curve and derivative profile *versus* temperature for unmodified Scots pine sapwood powders.

**Fig. 2:**
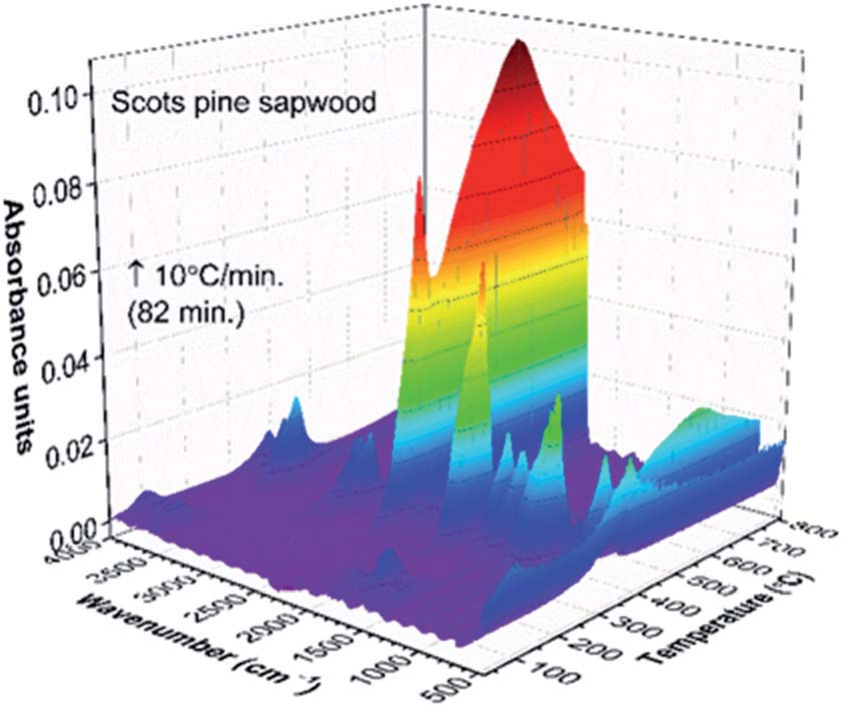
TG-FTIR absorbance spectra 3D stack plot of unmodified Scots pine sapwood pyrolysis components as a function of temperature.

**Fig. 3:**
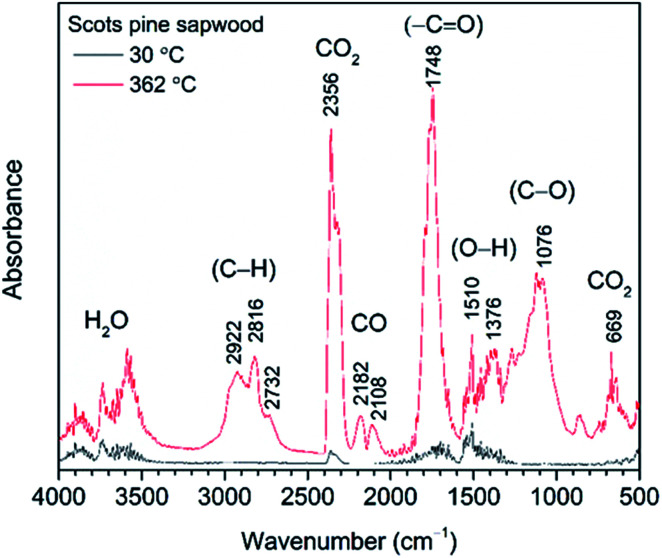
FTIR absorption spectra obtained at 30 °C and 362 °C (maxima weight loss during the pyrolysis) from TG-FTIR gas analysis of untreated Scots pine sapwood.

**Fig. 4:**
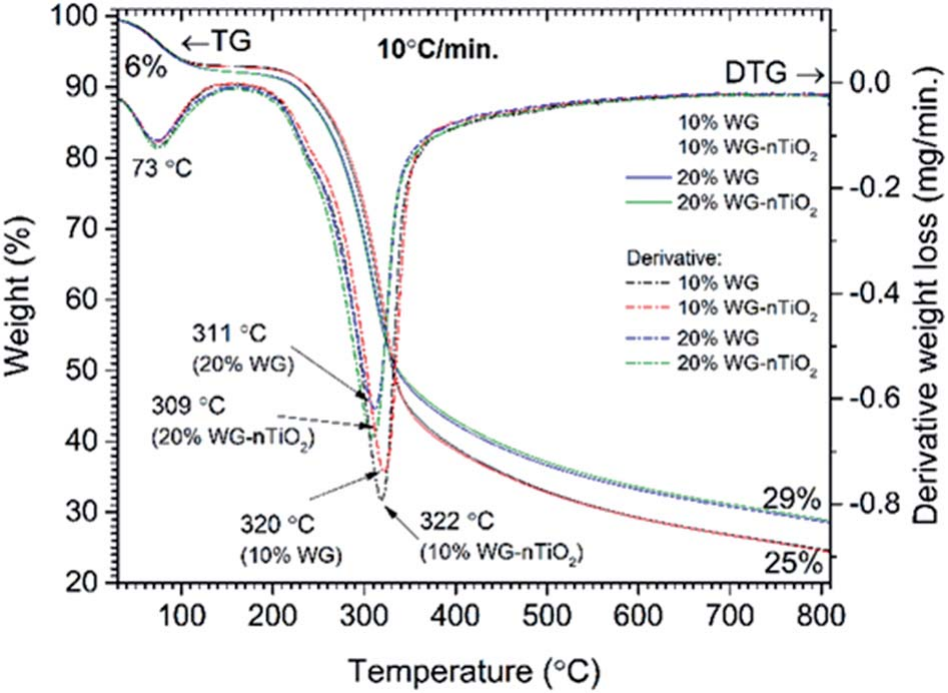
TG and DTG curves of pine wood modified with 10% Na_2_SiO_3_/-*n*TiO_2_ and 20% Na_2_SiO_3_/-*n*TiO_2_.

In addition, a citation to Fig. 12 (inset) on page 27982 of the original article should be corrected to refer to Fig. 13 (inset).

The Royal Society of Chemistry apologises for these errors and any consequent inconvenience to authors and readers.

## Supplementary Material

RA-009-C9RA90075F-s001

